# South Sudanese Refugee Survivors of Sexual and Gender-Based Violence and Torture: Health and Justice Service Responses in Northern Uganda

**DOI:** 10.3390/ijerph17051685

**Published:** 2020-03-05

**Authors:** Helen Liebling, Hazel Barrett, Lilly Artz

**Affiliations:** 1 Centre for Trust, Peace and Social Relations, Coventry and Warwick Universities, Coventry CV1 5FB, UK; 2 Centre for Trust, Peace and Social Relations, Coventry University, Coventry CV1 5FB, UK; H.Barrett@coventry.ac.uk; 3 Gender, Health & Justice Research Centre, University of Cape Town, Cape Town, Observatory 7925, South Africa; Lillian.Artz@uct.ac.za

**Keywords:** South Sudanese refugees, sexual and gender-based violence, torture, integrated service provision, Northern Uganda, health, justice

## Abstract

This British Academy/Leverhulme-funded research investigated the health and justice service responses to the needs of South Sudanese refugees living in refugee settlements in Northern Uganda who had been subjected to sexual and gender-based violence (SGBV) and torture. It involved the collection and thematic analysis of the narratives of 20 men and 41 women who were refugee survivors of SGBV and torture, including their experiences in South Sudan, their journeys to Uganda and experiences in refugee settlements, in particular their access to health and justice services. Thirty-seven key stakeholders including international, government, non-government organisations and civil society organisations were also interviewed regarding their experiences of providing health and justice services to refugees. All refugees had survived human rights abuses mainly carried out in South Sudan but some had also occurred on route to Uganda and within Uganda. Despite the significant impact of their experiences, the analysis indicated that there was limited service response in refugee settlements in Northern Uganda once the immediate humanitarian crisis ended. The thematic analysis indicated five main themes coming from the interviews. These included: the nature of refugee experiences of SGBV and torture, including domestic violence and child abduction and forced marriage; issues associated with service provision such as lack of adequate screening and under resourcing of health and justice services; a lack of gender sensitivity and specialist services, particularly for men; the sustained involvement of civil society organisations and local non-governmental organisations in providing counselling and offering emotional support and hope to survivors; and enhancing health and justice responses and services to improve refugee recovery, dignity and resilience. The authors recommend that integrated gendered and culturally sensitive service provision should be adopted, which brings together formal and informal health, justice services and survivor support programmes.

## 1. Introduction

South Sudan gained its independence from Sudan in 2011, but hopes of a peaceful future were thwarted when fighting broke out in December 2013. Since then, conflict has spread across the country and this has led to immense loss of life, dislocation of people, and land occupation. Almost 400,000 people have been killed and over 4 million people displaced [1]: 2 million within Sudan and a further 2.5 million as refugees to neighbouring countries [1]. Half of those refugees who have fled their homes now live across the borders in Uganda, Ethiopia or Sudan [2]. The South Sudan displacement crisis is now the largest in Africa and the third largest globally after Syria and Afghanistan [3]. 

By January 2016, over 644,000 refugees had fled into the neighbouring countries of Ethiopia, Kenya, Sudan, and Uganda [4]. Uganda had previously received a significant number of South Sudanese refugees, but in 2016 refugee settlements in Northern Uganda experienced a rapid influx of refugees displaced by the resurgence of violence. United Nations High Commissioner for Refugees (UNHCR) estimates that since the insurgence began, more than 290,000 refugees have been settled in refugee settlements in the Adjumani, Arua, Kiryandongo and Koboko districts [5]. The numbers are uncertain, as in 2018 Government of Uganda sources [6] reported to the authors that two refugee settlements (Bidi-Bidi and Adjumani) had taken in almost a half a million South Sudanese refugees in the 2016 crisis. The Real Medicine Foundation (RMF), the health care implementing partner for the UNHCR in Bidi Bidi since 2016, has provided healthcare services to 400,000 refugees and the host population, and Dan (Danish) Church Aid estimated that 3500 refugees came through a day in the height of the migration; from August 3rd to December 8th 2016, with 272,000 resettled in Bidi Bidi alone [7]. Twelve of Uganda’s 121 districts host the overwhelming majority of refugees. About 92 percent live in settlements alongside local communities, mainly in Northern Uganda or West Nile (Adjumani, Arua, Koboko, Moyo, Lamwo and Yumbe) with smaller numbers in Central or Mid-West Uganda (Kiryandongo and Hoima) and Southern Uganda or South West (Kyegegwa, Kamwenge and Isingiro). Urban centres, in particular Kampala, are home to eight percent of the refugee population [5].

Uganda is the largest refugee hosting country in Africa, home to nearly 1.45 million refugees, which represents 3 percent of Uganda’s entire population [5]. United Nations Children’s Fund (UNICEF) is supporting the Ugandan Government’s social mobilisation efforts and water, sanitation and hygiene interventions. Other organisations, such as the Real Medicine Foundation, offer healthcare services to 400,000 refugees and the host population, through the deployment of about 500 medical and support staff, offering comprehensive primary healthcare services, medical and nutrition screening of new arrivals, maternal and child health services, reproductive health services, HIV/TB care, immunisation and heathy living programmes (including malnutrition and disease prevention) [8]. Médecins Sans Frontières (MSF) offers basic healthcare for refugees at transit centres, as well as screening for malnutrition, outpatient and inpatient departments, maternity wards and an intensive therapeutic feeding centre [9]. Meanwhile, others, such as Samaritan’s Purse, offer awareness programmes about sanitation and hygiene, and War Child Canada provides legal aid and accelerated learning programmes (to ensure proper integration of refugee education into the Ugandan education curriculum). Nearly 34,000 South Sudanese refugee children are accessing formal or non-formal basic education through Early Childhood Development learning centres and adolescent learning programmes [5]. 

However, the level of funding for the refugee response in Uganda has recently reached an all-time low, with only 42 percent of earmarked contributions received as of October 2018 [5,10]. Thus, whilst the number of refugees per 1000 Ugandan inhabitants has tripled to 35 since 2016, putting a huge pressure on local resources and services, external aid has been dwindling over the years, causing major gaps in the service response to refugees in refugee settlements. Refugee response partners continue to face enormous challenges in stabilising existing programmes and often meeting the minimum standards of service provision, let alone investing in long-term and more sustainable interventions. 

Severe underfunding has particularly compromised the quality of child protection and education services and limited investments in prevention and response to sexual and gender-based violence (SGBV), environmental protection, support for host communities, and permanent community infrastructure. With 34 percent of its population living below the income poverty line (US$1.9 per person per day), a recent report argues that Uganda struggles to be able to fully realise a comprehensive refugee response and maintain its progressive refugee policy without adequate support from the international community [5,11].

The conflict in South Sudan is renowned for the high level of SGBV and torture perpetrated on civilians [10,12,13], with Zanaib Bangura, the UN Special Representative of the Secretary-General on Sexual Violence in Conflict, stating that the widespread nature of sexual violence in the South Sudanese civil war was the worst she had seen in almost 30 years of professional life [12]. The recent report by Justice Africa [13] makes the argument that whilst the horrific scale of civilian abuse at the hands of South Sudan’s warring parties has come to dominate commentary on the country’s conflict with SGBV described as a ‘weapon of war’ and labelled as ‘war crimes’ and ‘crimes against humanity’, this is a simplistic interpretation of SGBV in the country. The authors discuss the ways in which a focus on sexual violence by warring parties has been misleading in terms of the actual SGBV-related security concerns of South Sudanese families and communities as well as the realities of local level violence, such as child and forced marriage, domestic and intimate partner violence and marital rape [13]. A further paper [11] highlights the structural violence associated with the local South Sudanese economy of bride wealth and the accompanying commodification of feminine identities and bodies, as well as female bodies being regarded as a reward for soldiers and other combatants. The authors relate how abduction, rape and sexual slavery of women has become acceptable in the face of conflict and economic crisis [11]. Justice Africa concludes that SGBV requires a long term approach and recognises the cycles of violence it perpetuates [13]. The majority of refugees from South Sudan have experienced a multitude of human rights abuses including sexual and gender-based violence and torture [13], however, in-depth information regarding the needs and access to services including mental health provision for this population is lacking [14,15,16]. 

The current research therefore moves forward the existing knowledge regarding the in-depth nature and lived experiences of South Sudanese refugees and service providers of the health and justice service provision and responses for refugee survivors from South Sudan who have experienced SGBV and torture and are currently living in refugee settlements in Northern Uganda. It also makes recommendations based on its findings regarding potential opportunities, to address some of these challenges.

## 2. Materials and Methods

Following ethical authorisation to carry out the research granted by Coventry University, the University of Cape Town, the Uganda National Council for Science and Technology, Gulu University (Uganda) and the Office of the Prime Minister in Uganda, primary data collection took place in May and June 2018. In total, 61 refugee survivors of SGBV and torture came forward and gave informed consent and were interviewed for this research including 41 female refugees (15 in Adjumani and 26 in Bidi Bidi) and 20 male refugees (11 in Adjumani and 9 in Bidi Bidi). Refugee participants were recruited through the Refugee Welfare Councils in refugee settlements, as well as through non-governmental organisations working in Northern Uganda, and were interviewed using a semi-structured interview schedule. Each interview lasted between 60 and 90 min. The interviews were held in a space where confidentiality could be guaranteed in Bidi-Bidi, Mungula and Pagriniga refugee settlements. All of the interviews with refugees were carried out with the assistance of an interpreter. Interviews were tape recorded and transcribed verbatim, with written notes also being taken during the interview. At the end of the data collection process, three feedback meetings were held with approximately 130 refugees in Adjumani and Bidi Bidi to share and validate the research findings. 

Thirty-seven key stakeholders were also interviewed, including health and justice service providers. These included representatives from the UN, Ugandan Government, international non-government organisations, local non-government organisations, civil society organisations and refugee organisations, providing security, health and justice services for South Sudanese refugees living in settlements in Northern Uganda. A choice of focus group interviews and/or individual interviews was given, and these were conducted in the work places of the participants in Adjumani and Bidi Bidi refugee settlements, as well as in Kampala, in May and June 2018. Each interview lasted between 50 min to 90 min. These were conducted in English. 

The data collected from refugees and key stakeholders were investigated using thematic analysis to identify key themes [17]. 

## 3. Results

The five main themes to result from the analysis are shown in Table 1, which also shows the sub-themes emerging from each of the main themes.

A thematic analysis of the interviews identified five themes that were related to important aspects of male and female refugee survivors’ access to, and the key stakeholders’ experiences in providing support and services for refugees. These are detailed in Table 1 and include: the nature of refugee SGBV and torture experiences; service provision; gender issues; and the involvement of civil society organisations (CSOs) and local non-governmental organisations (NGOs). The fifth theme of improving health and justice responses arising from the data analysis is incorporated into the discussion section below. 

### 3.1. Nature of Refugee SGBV and Torture Experiences

#### 3.1.1. SGBV and Torture

Service providers, as well as refugees, reported that men, women and children were subjected to SGBV, which they believed was used as a ‘weapon of conflict’ and ‘torture’ in South Sudan. This included torture from Government forces as well as rebels, for example being burnt on the face and head with a red hot knife or metal instrument. Seventy-five percent of the men interviewed reported being beaten and/or tortured, with 25% claiming they had been shot at by rebels or had been sexually assaulted/raped. Women and children were raped and gang raped and men were raped and sexually assaulted as described by a local government official:

“The fighting in South Sudan is very complex. Sexual harassment and SGBV is rife in South Sudan; SGBV is used as a weapon of war, even small girls are gang raped and as a result are crying. Children, women and men are raped. It causes lots of emotional distress and the whole family is affected. Survivors are often raped in front of family members. To those forced to witness it is psychological torture.”

Service providers expressed their view that the majority of human rights abuses, including torture and SGBV, took place in South Sudan. A male refugee in Adjumani describes the situation that many have escaped from:

“Most refugees have been captured and tortured in South Sudan and held in military barracks. There is extensive torturing and robbing and anyone could be a rebel. Most of us were threatened with our lives and experienced atrocities, for instance removing our finger nails, tying penises, being shot; even girls were tortured and shot.”

One woman interviewed in Adjumani described what happened to her:

“I was selling beer in Juba…. The attackers wanted to kill me so they could take away my children. They tied my legs up, head down, and beat me between legs, the head and left me unconscious thinking I was dead. I was left there until morning. Then, my husband took all [the] children. The neighbours came, helped me down, gave me water, and took me to the health centre in Juba.”

In Bidi-Bidi, a male refugee recounted his experiences in South Sudan:

“…when I was in South Sudan, the soldier services have arrested me, they tortured me, they bite me, and now I am feel in pain, my neck here is in pain, and even my head, I just feel in pain. I was put inside the throne for one week (tied to a chair), outside under the sun.”

A male survivor in Pagrinya tells a similar story: “They torture me (burned me on foot), hit my chest and even performed homosexual acts on me.”

Refugees with bullet wounds feared coming forward for treatment, due to concern they could be labelled as ‘rebels’. Women and girls frequently became pregnant following rape on their journey to the settlements and had to suffer the stigma and discrimination this brought with it. Male and female refugees were often unable to work due to their injuries, which included genital damage amongst both men and women following SGBV, as well as disability due to torture and psychological trauma. Whilst most SGBV and torture had taken place in South Sudan or on the journey to Uganda, there were reports of SGBV taking place in refugee settlements in Northern Uganda, mirroring the culture of SGBV in South Sudan [11,13]. It was reported by both service providers and refugees that appropriate health and justice services that took into account the culture of SGBV amongst South Sudanese communities were an essential part of refugee recovery and resilience.

#### 3.1.2. Child Abduction and Marriage

Justice Africa [13] report that economic hardship and the civil war in South Sudan have ‘boosted’ an already dowry-dependent local economy, in which parents view their daughters as their only source of wealth (their ‘bank accounts’). This culturally sanctioned view of girls and women as commodities and the associated SGBV violence has accompanied refugees who are living in Uganda. A women refugee in Adjumani explained:

“Children are being abducted: somebody is trying to take the 13-year-old daughter of my deceased sister. My sister stated in her Will that her child should be looked after by me. My sister married a Dinka (a different father to the 13-year-old girl) and had a son by him who subsequently died. Her husband killed my sister in revenge as he blamed her for the son’s death. Now her husband in South Sudan wants to take the 13 year-old-girl. I think it is for marriage. Such situations are common in South Sudan. There are lots of refugee children in the protection unit in Adjumani. I feel insecurity is caused by ourselves as habits from South Sudan are brought into the settlement including abducting children as revenge. This is a characteristic that takes time to change and there is a big need for education.”

Within the settlements personal security was reported to be lacking, and particularly women and girls continued to experience SGBV, including the abduction of young girls from Uganda into South Sudan for the purposes of child marriage. Such abductions were reported to be mainly perpetrated by family members, including fathers. It was explained that this was partly due to a clash of culture and differing legal frameworks between South Sudan and Uganda. Disparities in Ugandan and South Sudanese marriage law created confusion and conflict, as in South Sudan a child can be married at puberty, whereas in Uganda 18 years is the legal age for marriage. Service providers were very conscious of the situation and were involved in awareness raising campaigns within refugee settlements to inform refugees that marriage of a child under the age of 18 is illegal in Uganda. One local official said:

“Refugees must live within Ugandan Law. In Uganda girls cannot marry before 18 years of age but in South Sudan it is 12 years. It is contradictory. If a girl is married under the age of 18 years in Uganda then fathers will be charged. This can cause conflict. Hence many girls are taken to South Sudan to be married under the age of 18. Lutheran World Federation is educating the South Sudanese refugees regarding the differences in the laws in Uganda”

Our research found that the police would intervene if a child at risk was reported to them. However, refugees testified that the perpetrators were often residing in South Sudan and would cross the border to abduct young girls for forced marriage. This makes it a crime that is very difficult for the Ugandan authorities to tackle. 

#### 3.1.3. Family Conflict and Domestic Violence

Domestic violence was reportedly very common in the settlements, reflecting the culturally accepted violence in South Sudan communities and families [11], but many women claimed it was worse in the refugee settlements. One reason given for this was the change in power relations between husbands and wives following refugee settlement. Male refugees had very often sent their female relatives with their children to the settlements ahead of themselves. As a result, the UN registered these women as the head of the household. Not surprisingly, it was reported that 70% of households in Adjumani and Bidi-Bidi refugee settlements were female headed. This had a positive and empowering impact on these women, and meant that within a refugee context these women were the main decision-makers for the family and received food rations. However, many husbands and male relatives of these women found this situation very difficult to accept. 

Service providers, including a UN agency and a local government official, confirmed that it was possible to change the name of the registered head of household at the request of the family members and in cases of family separation. However, this could only be done if the proposed head of household was a registered refugee. As many male refugees failed or refused to register as refugees when they joined their families, this was not possible and compounded the situation as household rations then had to stretch to feed unregistered household members, often causing conflict and shortages within the family unit. Many refugees cited this change in family power dynamics, as well as substance abuse, as reasons for the high levels of domestic violence in the refugee settlements. 

The research found that men were very reluctant to report domestic violence perpetrated on them by women. A member of the police force stated:

“My understanding is and what I have found out from South Sudanese refugees is that when women beat men and you come to report it degrades like you are not a man. I always advise that when it happens you have to report and it is not that all the cases should be taken to Court as we also do counselling”.

It is therefore likely that the number of domestic violence cases is under reported, with many suffering in silence or getting help through informal mechanisms.

### 3.2. Service Provision

#### 3.2.1. Screening

Service providers discussed the fact that refugees were frequently not screened on arrival in reception centres in Uganda or in the refugee settlements they are allocated to. This is despite the fact that a screening tool had been developed by the Refugee Law Project [18]. It was suggested that all refugees should be screened regarding their experiences in South Sudan, on their journey to Uganda and in Uganda, so that appropriate services could be provided. A representative of the Refugee Law Project stated:

“If you go to reception points, they do an incredibly shallow kind of screening and it’s just absolutely hopeless. So, you know we would still advocate for across the board screening and recognising that the dynamics are extremely complex and that what works at month 1 might be different to 6 months and what works 5 years after”.

The Refugee Law Project and other stakeholders argued that screening needs to be conducted on arrival, and should be undertaken at regular intervals, as the needs of refugees change over time. Both service providers and refugees discussed the importance of registration, as it is only with registration that refugees can access health services, register at schools, and benefit from development projects (e.g., housing).

#### 3.2.2. Health Services

“The treatment is not very good here because the health system is out of date. I was taken to the clinic as one of my ribs was broken and nothing was done. Now it is pointing the other way. There is no proper medication. I am requesting to be taken to another hospital or for doctors to be sent to help me”

(Male refugee, Bidi Bidi)

Health service facilities, including the availability of drugs and other treatments in the refugee settlements, were reported by key informants as being inadequate and unable to respond to refugees’ needs, with shortages in HIV, malaria and other life-saving drugs. There were Health Centres 1, 2 and 3 in the settlements but a lack of Health Centres 4. Health Centres 1 offer the most basic level of care (basic injury treatment, over-the-counter medicine distribution and referrals), and so according to the Ugandan government’s health policy [19], every district should have at least a Health Centre 2. A Health Centre 2 facility, normally serves a few thousand people and should be able to treat common diseases like malaria and provide antenatal care. They are normally nurse led. A Health Centre 3 facility should be found in every sub-county in Uganda. These centres are led by a senior clinical officer and run general outpatient clinics, a laboratory and have a maternity ward. Within the refugee settlements, there is severe understaffing and resourcing of these clinics. A Health Centre 4 serves a county or a parliamentary constituency and is a mini hospital. It should have the kind of services found at Health Centre 3, but also should have wards for men, women, and children and be able to admit patients. It is supposed to have a senior medical officer and another doctor, as well as a theatre for carrying out emergency operations. No Health Centres 4 were present in any of the refugee settlements where this research was carried out. Refugees requiring such treatment had to access Ugandan government hospitals outside the settlements as far afield as Gulu and Kampala.

Refugees and key informants informed us that within the settlements’ Health Centres there was a lack of specialists, including reproductive and gynaecological professionals to assess and treat female survivors of SGBV and torture. Maternal health care was also reported as inadequate. Specialist professionals for screening and treating male refugee rape survivors were also lacking. A female refugee explained the gaps during a feedback focus group in Adjumani:

“We need specialists including gynaecologists and midwives needed to deal with our health problems and treat them. We also need psychological counselling and peer support.”

The Refugee Law Project provides medical treatment and some counselling for male refugees that they have found has increased disclosure by male survivors of SGBV [18]. There were other non-government, government and international organisations that provided some services that included assessment and treatment. An interview with a representative from the UNHCR said they provided:

“Transit centres with medical teams attending to new arrivals. Identification of serious cases and treatment is provided. There is documentation of some health-related issues and violations”.

The research found there was limited provision of counselling for refugees and only one psychiatric clinical officer in one of the two settlements (Adjumani and Bidi-Bidi). One female refugee we interviewed in Adjumani, following being raped and conceiving a child, narrated that she was taken by Lutheran World Federation to the health centre and was supported with clothes for her baby, but was not offered any counselling. Refugees and service providers informed us that the counselling provided was limited and tended to be based on cognitive-behavioural therapy (CBT), with a lack of the person-centred, empowering and holistic approach required [20,21,22,23]. During an interview with the Danish Refugee Council, who supported groups of female survivors of SGBV, we were told that:

“Women are informed regarding referral pathways for basic counselling. Those survivors who have mental health trauma or reproductive health needs are referred to our partners including Real Medical Foundation, Transcultural Psychosocial Organisation, and Medicines Sans Frontiers etc…”

Health centre provision was supplemented by civil society organisations who offered some limited health screening for refugees, for example for HIV and TB, and health interventions. Some civil society organisations also worked together with traditional structures, such as refugee welfare councils and faith-based organisations, to provide community outreach regarding education about mental health and physical health problems. However, there were many challenges providing even adequate services for refugees. A male refugee in Adjumani sums up the situation:

“There is a Health Centre 2 and 3 in Adjumani but the Health Centre 2 here has no admission facilities. The Health Centre 3 cannot manage the health needs of refugees as they are over-stretched and for survivors of torture and SGBV we really need to go to Adjumani, Gulu or Kampala and UNHCR are supposed to pay. However, sometimes refugees are transferred for treatment and we are told the money has not been paid. There is no psychiatric clinical officer, no HIV+ medication. Language barriers are also a problem and sometimes health staff demand for money.”

#### 3.2.3. Justice Services

Survivors and key informants reported that the criminal justice system failed to provide adequate justice for refugee survivors of SGBV and torture. Services were delivered by the police, UN organisations and civil society organisations, including faith-based organisations. A few local NGOs provided legal aid, support or facilitated access to the formal justice process, counselling and psychological support. Access to the formal criminal justice process, such as the police and magistrates’ courts, was reported by refugees to be lacking, with refugees settling criminal matters through informal community structures, which many refugees considered a legitimate dispute mechanism.

All the survivors living in the refugee settlements in Uganda that participated in this research confirmed they knew and understood the criminal justice reporting system of alleged crimes that took place within the settlements. Whilst they all knew of the (formal criminal justice) reporting system for such incidents, they questioned the effectiveness of the process at times. One female refugee from Adjumani who reported an incident of SGBV in the settlement to the police told us:

“The only problem is when I reported to the police, they asked for money, saying if I don’t have money they are not going to investigate this issue”.

The issue of bribery is taken seriously by the authorities, with supervision structures being put in place by stakeholders with the aim of tackling this issue. Those who spoke of sexual offences in the settlements said that there was a clear health pathway for cases, but these were rarely followed through effectively by the formal criminal justice process. Access to justice was reported to be particularly problematic for women, as the courts were often long distances from the settlements and there was a backlog of cases, with 10% of cases taking over one year to be heard. According to one of the service providers interviewed in Bidi Bidi, and as recently documented, mobile courts have been introduced in all zones of the Bidi-Bidi Settlement to try to reduce waiting times and overcome the logistic challenges faced by refugees [24]. However, none of the refugees interviewed were aware of this service. Most male refugees opted not to report crimes committed against them, often due to high levels of stigma and shame, something, of course, that is not unique to refugee settlements or the Ugandan context.

As a result of these issues of access to the formal criminal justice process, many refugees reported that they used informal mechanisms to resolve differences. These informal structures comprised mainly customary courts or families, resolving disputes through trusted intermediaries [13]. Such informal processes are criticised by experts [11,13] for concentrating on social reparation rather than justice for the individual. In cases of SGBV involving unmarried girls or women, the outcome is usually either arrangements being made for the victim to marry the perpetrator, or the perpetrator paying ‘damages’ in lieu of the reduction/loss of dowry, to the victim’s family [13].

The majority of refugees told us they generally felt secure in Uganda. However, they remained concerned about violence and SGBV in the refugee settlements. Female survivors were concerned about their personal security, their vulnerability to SGBV (by intimate partners, family and the local population), and the abduction of their daughters for child marriage. Service providers informed us that there was security in the settlements provided through neighbourhood watch and police posts in all zones with armed forces. Social protection was also provided by community organisations in conjunction with the Office of the Prime Minister and UNHCR. However, some of the refugees reported they had witnessed, or experienced, violence in the settlements; some of this as a result of ongoing political or family conflict originating from South Sudan, some relating to insurgents crossing borders for supplies, and some as a result of community-based crime. Service providers testified that they worked closely with traditional leaders and Madi chiefs, including the Customary Courts, on transitional justice issues. Psycho-education groups were held with female refugees to educate them regarding access to justice. The police had several challenges to providing effective services including lack of staff, particularly female police officers (although this was being addressed in Bidi-Bidi) and logistical resources to carry out their role effectively, such as a lack of transportation to reach reported incidents. A police officer interviewed describes some of the difficulties faced in providing justice for refugees:

“When they commit offences here they run to Sudan because they fear to be arrested or detained, so they first run to Sudan. The challenges we also face is when a person commits an offence, when he is aware this has been brought to the attention of the police he becomes very arrogant and fights the police. When you go for the arrest, parents can refuse for the perpetrator to be arrested and sometimes they are serious cases, the offences they have committed, and they say that they have their local court.”

It is evident that formal (state) policing and magistrates’ courts are operational in the settlements and that refugees, out of necessity or partiality, also utilise local customary structures. The settlements are not unique in their shortage of visible police, skilled investigating offenders, and the backlog in court cases. There does, however, seem to be a heightened resistance to reporting SGBV to the police or state structures, for fear of making an already challenging social context, more pronounced.

#### 3.2.4. Other Services Provided

The Ugandan Government treats South Sudanese refugees as a prima facie case, therefore they do not need to apply for asylum status and thus, few formally document their human rights abuses that they have been subjected to in South Sudan. The Ugandan government allocates a plot of land (30 metres squared) and farming implements to refugees. Further support is given to those refugees who are considered ‘vulnerable’. According to UNHCR, this includes those refugees who are vulnerable due to their situation, which include circumstances on route that result in vulnerability and those who are individually vulnerable and who have characteristics which place them at particular risk e.g. children, particularly those who are unaccompanied or have been separated from family; older people; those with mobility, sensory, intellectual, or other disabilities; those with chronic illnesses or other medical needs; survivors of trafficking who do not fall within the scope of the refugee definition; and as particularly significant for our study, those refugee survivors of torture or trauma on route [25]. This document goes on to state that the human rights of migrants in vulnerable situations need to be respected, and their immediate and specific needs met, including through rescue; appropriate reception arrangements; family reunification; access to medical assistance, including psychosocial services; and help in availing themselves of national or consular services. UNHCR’s recently updated 10-Point Plan in Action on refugee protection and mixed migration brings together a range of tools and practices for timely and effective responses, which includes a focus on refugee survivors of abuse or trauma [26]. Service providers we spoke to also referred to English language services provision, socio-economic activities for refugees and livelihoods training including forestry. Economic activities were reported by refugees only to be allowed within the refugee settlements. However, the Ugandan government faces many challenges in providing adequate services for the huge numbers of refugees living there, as explained at an interview with a member of local government:

“Resources are limited 7% of the refugees needs are met but 93% of refugees needs are not funded and we cannot meet their needs and the environment is being destroyed. There are no adequate health services and the refugee population puts a lot of pressure on our existing services and facilities including lack of treatment for HIV infection”.

### 3.3. Gender Issues

#### 3.3.1. Lack of Gendered Understanding

It was generally felt by service providers that services more responsive to the unique needs of refugee men and women and their living contexts, such as sexual and reproductive health services, support for female-headed households, land skills development projects and justice responses that are sensitive to complexities of SGBV cases, were needed with respect to health and justice provision. It was reported that there was a lack of a gendered understanding of the needs of refugee survivors. A representative of an NGO stated:

“There are several factors triggering gender-based violence including the selling of goods by men, women gaining their rights, the accumulation of problems, stress and distress, the inability to send children to a good school, loss of properties and businesses and an inability to receive treatment for a chronic illness.”

#### 3.3.2. Need for Gender-Informed Specialist Services

An analysis of the interviews also indicated the pressing need for gender informed specialist services. SGBV services were often viewed as being accessible to or focused on the experiences of women, excluding men who also experienced SGBV and torture both in South Sudan and within the settlements. A female refugee in Adjumani described the difficulties men had in disclosing abuse by women:

“Men fear to open up and many are tortured by women.”

As this short quote illustrates, it was felt that men found it harder to disclose abuse and mistreatment, and providing medical treatment and support (for them specifically) assisted men to overcome stigma and shame and come forward [18]. 

Key stakeholders also emphasised the need for a greater focus on tackling the sexual and reproductive health needs of women who found their health problems stigmatising, and therefore did not come forward for treatment. A representative from the Refugee Law Project stated:

“Amongst the 30% that we screen who need to go to hospital we will find the woman who was gang raped 20 years ago and is still oozing god knows what because she never got the right treatment, so she has infections that have never cleared up, literally. And then open wounds and somehow it’s all interconnected. All oozing to the point where she stinks and her own family can’t have her in the same hut as them. Why is that happening? I mean it’s just unbelievable. But it’s true.”

What is clear is that the conflict in South Sudan, the migration to Uganda, and settlement exposed men, women and children to all forms of sexual offences, physical violence and torture. Gender, in this instance, was not a discerning factor. However, service provision has not fully recognised the issues experienced by males, and thus services for men are lacking or under-resourced. 

### 3.4. Involvement of Civil Society Organisations (CSOs) and Local NGOs

#### 3.4.1. Provision of Emotional Support

There were a number of local civil society organisations (CSOs) and NGOs supporting refugees and providing psychosocial services to refugee survivors of SGBV and torture, including faith-based organisations and church personnel. A faith-based organisation provides a typical example of the services provided:

“Samaritan’s Purse is a church organisation which provides Bible based trauma healings, spiritual and counselling services. Samaritan’s Purse provides the survivors’ host communities with a package from the Bible Society of Uganda and America. They conduct training for adults and children on Bible based trauma”.

CSOs provided psychological support for refugee survivors through groups and individual counselling and worked with all faiths regardless of their own denomination. One trauma counselling organisation explained:

“We work closely with traditional leadership structures in the villages including the community leaders and faith-based leaders for our interventions. We train refugee mobilisers in the communities and use Biblical principles. We are a Christian organisation but we also work in Muslim communities.”

#### 3.4.2. Instil Hope

It was felt that CSOs, local NGOs and faith-based organisations provided an important role in assisting to instil hope among refugees. Psychologically being able to build trusting relationships following traumatic experiences with a trusted confidant, including friends and families, as well as individuals in organisations, is an important part of processing and healing traumatic experiences and building resilience in refugees [27]. Refugee survivor groups were formed in the refugee settlements by such organisations and were reported by refugees to be very helpful for connecting with others who had similar experiences, validation, psycho-education and sharing [28]. A male refugee from Bidi Bidi described the support he and his family received from a church and stated:

“I didn’t receive any help from any organisation but the neighbours consoled me. There were also some tapes provided by the Baptist church, which, preach New Testament Bible in my local language. When the images flashbacks of torture come, I just switch on the tapes and this helps me. As family, we also listen together and go to the Catholic Church on Sundays”.

CSOs, including faith-based organisations (FBOs), through their work, are also engaged in raising awareness regarding the dangers of SGBV in the settlements and also provided training for the police. CSOs and FBOs worked closely with community and traditional leaders to increase the community impact of their work. This included training traditional leaders to act as refugee mobilisers within their communities.

It was felt by some we spoke to that the contribution of CSOs and FBOs was sometimes not well recognised by the UN, Ugandan government and international NGOs, and that FBOs lacked sufficient funding to support survivors effectively. CSOs were working together to try to boost the capacity, to provide services for survivors and to increase referrals.

The research indicated that as well as building refugees’ own resilience, there could be more scope to further integrate CSOs and local NGOs into psychological health and justice services, as indicated by the previous quotation, as they were sometimes able to build trust with South Sudanese refugee communities, where attending church for instance, can be important, and are also aware of local cultural and political sensibilities. A local NGO providing psychosocial support and counselling described some of the barriers they faced in providing support to refugee survivors in the Bidi-Bidi Settlement:

“There is an overwhelming demand for the services by SGBV victims and inadequate resources and understaffing. Due to cultural sensitivities there is a failure to share the problems affecting both men and some women. It is hard for us to access some locations as there are large zones with inadequate transport. Zone 5 had new arrivals in 2016 and therefore has a high demand for medical treatment. There is a lack of urgently required drugs in Health Centres 2 and 3; particularly Post-Exposure Prophylaxis (PEP) and medication for unwanted pregnancies”.

## 4. Discussion

The research carried out found that all refugee participants reported to have experienced or witnessed one of more of the following human rights abuses: violence (including beatings, being shot), sexual and gender-based violence (SGBV), physical and/or psychological torture and other human rights abuses, such as being unlawfully detained, being robbed, or being denied healthcare. Men reported more cases of violence and physical and psychological torture and fewer cases of SGBV than women, whilst women were subjected to high levels of violence, including SGBV, but less of torture. Seventy-five percent of male refugees interviewed reported being beaten and/or tortured, with 25% claiming they had been shot by rebels or had been sexually assaulted/raped. The majority of female refugees participating in the research had experienced SGBV either from South Sudanese Government soldiers and/or rebels, as well as from their husbands and other family members, particularly after arriving in Uganda. 

When survivors had access to health services, it was mainly for women who had experienced SGBV, pregnant women or those with minor aliments or injuries. Everybody involved in the research agreed there needed to be more focus on tackling gynaecological health problems of women who found these issues stigmatising, and therefore did not come forward for treatment. None of the male refugees had sought testing or treatment for SGBV including rape. All the men interviewed stated they had not reported their injuries officially and generally the only treatment available was paracetamol medication.

We were informed by service providers that SGBV counselling services were available to both men and women who were identified or reported cases, although the refugees we spoke to told us that they rarely had access to these services. Whilst the majority of female refugees interviewed confirmed they had participated in more than one counselling session, sixty-five percent of male refugees had not received any counselling. Much of this counselling was provided by people who had no formal training. It was also notable that there were limited counselling services available for those refugees who had experienced torture. In terms of the impact of traumatic experiences on South Sudanese refugees’ emotional health, a study [4] in conjunction with the Peter Alderman Foundation concluded:

“The types of mental health and psychosocial problems among South Sudanese refugees in Northern Uganda were diverse and the burden appears to be considerable, yet there are currently few available services. The assessment indicates the need for a range of services addressing social concerns as well as varied types of mental conditions.”

One evaluation of Tutapona’s trauma counselling services demonstrated the combined positive impact of psychosocial services and CBT education on trauma, which increased refugee empowerment, resilience and well-being [28]. The psychological effects of SGBV and torture on the male and female refugees we spoke to affected individuals and communities as a whole. The unaddressed consequences on these survivors are disabling and beyond what existing local services can cope with. Our research supports earlier work that argues it is highly important to focus on what refugees can do to help themselves to cope with their predicaments. Human resilience and the ability to recover after traumatic events should not be underestimated. Besides the provision of counselling and psycho-education support or therapies that might be required for specific refugees, there are many things that can be done at personal and community levels to help refugees adapt, increase their well-being, regain confidence, dignity, and the feeling of being in control. The role of refugee survivors in self-managing their psychological well-being needs to be better understood and encouraged. This gives further support that an integrated approach is required for refugees in these settings [29].

There was no mechanism for refugees to report atrocities that had occurred in South Sudan or during their journeys to Uganda. For alleged crimes committed within Uganda, access to formal criminal justice was also reported by refugees and service providers to be lacking, with refugees often having to settle criminal matters through customary structures. Refugees were not aware of a clear pathway for accessing justice services and criminal incidents were rarely followed through effectively. Most male refugees opted not to report crimes committed against them, often due to high levels of stigma and shame and a lack of service responses for their needs. It was generally felt that more sensitive approaches towards gender differences of refugee survivors were needed. Male survivors were often excluded from SGBV programmes and men found it difficult to discuss domestic violence perpetrated by women in the settlements and abuse and torture they had experienced. Research has argued that in cultures where traditional gender roles are strongly adhered to, male sexual torture survivors were most likely to suffer in silence due to the stigma. Likewise, some helping professionals internalise this societal stigma and thus do not believe that men can be sexually tortured, as one report highlighted by stating: “one therapist said that she had not believed that men could be raped until one night a man was brought in naked and bleeding from the anus.” [30]. As a result, such attitudes from helping professionals further discourage victims of sexual torture from seeking help and justice. The Refugee Law Project provided some specialised medical treatment specifically for men. They found that this assisted male survivors of rape to overcome stigma and shame, and therefore discuss their experiences of sexual violence and torture [18].

Child abduction and forced marriage out of the settlements to South Sudan was identified as an issue by all key stakeholders and many refugees. UNHCR and the Office of the Prime Minister (OPM) had provided public posters warning about child marriage and child trafficking to inform refugees that this was unlawful in Uganda. However, in all of the Commander’s Compounds we visited, there were posters displaying pictures of children who had ‘disappeared’, demonstrating how difficult it is for the authorities to tackle this crime.

The results of our research suggest three areas that require further consideration, which provide evidence and strong arguments for an integrated approach for addressing the health and justice needs of South Sudanese refugees in Northern Uganda to be implemented.

- Screening Which Integrates Health and Justice Issues:

The research argues for more collective and collaborative work for better and widely acceptable screening, for different forms of GBV, which encompasses SGBV and torture, and acknowledges the gendered aspects of these abuses and includes both physical and emotional components including whether the survivor feels they have received justice (formal or informal) closure. A more systematic rigorous approach to screening and documenting refugees’ SGBV and torture experiences would help ensure that the prevalence and nature of SGBV, that unfortunately is widespread amongst refugees, can be better identified, reported, redressed and treated than is currently the case [31,32,33]. Indeed, internationally there is a lack of data on the prevalence of SGBV and torture amongst refugees, which requires attention [34]. Inevitably this impacts on resource provision and the effectiveness of service planning in this area. Our research extends the current evidence base by arguing for an integrated gendered, culturally sensitive and rights approach described in the section below to understanding refugees’ experiences of SGBV and torture and responding to the impact of these atrocities, as both have to be addressed simultaneously to obtain the best outcomes for refugees [14,15,20,21,22,23].

All the refugees and service providers involved in this research appreciated Uganda’s humane policy and approach towards refugees, and felt services were doing their best in a challenging context [35]. However, we found the lack of an integrated approach to address refugees’ health, social welfare and justice needs was undermining refugees’ path to recovery, resilience and dignity. The authors note that there are considerable gaps in evidence for what responses, approaches to treatment, and other support, including informal mechanisms, are effective in assisting refugees who have experienced SGBV and torture. An evaluation of social enterprise groups established for the same refugees interviewed in the current project, through Coventry University funding obtained by the authors, finds support for integrating a social enterprise model which increased refugees’ ability and skills to income generate, their resilience and emotional wellbeing [14,15].

- A Culturally Sensitive Gendered Approach:

The research findings provide further evidence for integrated gender and culturally sensitive services that focus on the gendered needs of male and female refugee survivors, whilst also accounting for timing and context of abuses, as well as the influence of stigma and shame on reporting and access to services. In terms of what we mean by this, we recommend a culturally informed health and justice care delivery; also termed culturally competent or culturally sensitive care [36,37]. This refers to the ability of a health and justice care practitioner or stakeholder to acknowledge their own cultural backgrounds, biases, and professional cultural norms and to incorporate relevant knowledge and interpersonal skills related to the care of refugees from different cultural backgrounds. This also involves being familiar with the health and social issues related to the experience of individual refugees including cultural, religious, social, and gender factors that come into play in the negotiation and implementation of treatment plans for a variety of health and justice issues, including, specifically, the gendered impact of torture and trauma. We recommend integrating a gendered approach which addresses and mainstreams gender and power inequalities, and recognises cultural and patriarchal mechanisms that perpetrate SGBV and challenges these using an intersectional approach [11,13,38]. We envisage that this includes the practice of recognising the inherent power imbalances between men and women, and taking into account these gendered implications in the development, implementation, and evaluation of policies and interventions for refugee survivors of SGBV and torture [39]. Accordingly, future NGO programming needs to respond to the relationship between female and male refugees’ resource/income access, and their resilience and agency including their sexual agency [13]. 

To expand on the recommended approach, firstly, our research finds that men and female refugees all experienced a range of SGBV and torture experiences, but their responses to these experiences were different. As a result we recommend agencies and services supporting refugees integrate a gendered perspective throughout their work, including an analysis of power relations, and the impact of this on the success of interventions as others has also supported [11,13,36,38,40]. Secondly, a focus on understanding women’s and men’s lives, the structural mechanisms perpetuating SGBV in South Sudan, including the dowry system, and the gendered power dynamics, which lead to unequal outcomes for women and male refugees. SGBV and torture and fleeing from their home gave rise to very different opportunities for refugees and varied methods for obtaining resources crucial to survival for men and women. Another constant for women in crisis and stability is the continuing issue of unequal gender power relations in conjunction with norms of female and male sexuality [11,13], as well as the influence of culture which others have argued have intensified men’s control of women and gender inequalities amongst South Sudanese refugees [11,13]. Thirdly, we argue that the responses for men and women refugee SGBV and torture survivors requires an in-depth and gendered analysis of the post-conflict state and its political terrain, its capabilities, functions, and cultural legacies. Fourthly, it requires health and justice service provision that conceptualises sexual and gender-based violence and torture as gendered, and draws on a resilience and empowerment framework for understanding and responding to refugee survivors needs [36]. 

Our findings lend further support to research [40], which argued that stigma and shame affect the ability to communicate SGBV and torture experiences, and then result in reduced access to specialised health services, lack of validation of experiences, lack of understanding by service providers and reduced ability to report abuses. Recent research has asserted that refugee survivors tend to have their human rights compromised or denied, and that they are at increased risk of SGBV and to receiving inappropriate responses to it [33]. Female refugees in our research were more likely to discuss their SGBV experiences than male refugees, and the gendered differences and impact of stigma and shame needs further attention, training and sensitivity by service responders and stakeholders.

In terms of access to justice services, our research found that refugees remained concerned about continuing violence and SGBV in the refugee settlements and women recounted incidents to us. Whilst they all knew of the criminal reporting system for such incidents, they questioned the effectiveness of the formal justice process. For this reason, female refugees said they would often opt for family reconciliation or interventions through informal community dispute mechanisms, such as customary courts, as has been found in other reports [11,13], rather than reporting domestic violence or SGBV to the authorities. Male refugee participants related that, while they were reluctant to report domestic based incidents, they did report instances that related to external threats arising from alleged perpetrators from South Sudan entering the settlements. Our research confirmed the need for more services for male refugee survivors as well as male and female torture survivors, and the need to consider a broader focus on informal and customary justice mechanisms. The continuing levels of SGBV in the settlements support other research carried out, that argues that the lack of accountability for acts of sexualised and gendered violence in South Sudan contribute to what has been referred to elsewhere as “cycles of violence within violence” where one form of violence begets another. The complex nature of the culturally, politically and socially embedded violence and atrocities in South Sudan against men, women and girls needs to be understood in terms of the continuing SGBV and related atrocities within Uganda, where the structural reasons for continued SGBV violations including the commodification of feminine identities and bodies, needs to be addressed to tackle the problems refugees continue to face [11,13]. We agree with previous work that recommends developing accountability mechanisms that cater to all experiences of injustice, to ensure that survivors of sexual violence can seek redress, interrupting the cyclical relationship between impunity and continued violence. As one of these recent reports recommends [13]:

“International partners need to work closely with local civil society to contextualise sexual violence and locate areas of compatibility between local cultures and customs and international human rights frameworks as entry points for programming that aims to empower and protect girls and women”.

- Involvement and Empowerment of Refugee Survivors:

Our research demonstrates the importance of involving and engaging with refugees in a meaningful and systematic way, in discussions of current and future service provision within settlements that is responsive to their changing needs. This would enable a more nuanced and gendered approach to refugees than is currently available, and also further empower refugees themselves. Refugees valued the opportunity to narrate their experiences in the current research and found this validating both in terms of their health and as an important form of informal justice recovery. Greater involvement of CSOs and local NGOs in supporting refugees’ own solutions would be a starting point for this process [29]. 

The research emphasises the benefits of the importance of listening, validating and documenting refugee experiences on a regular basis in order to provide more culturally sensitive, gendered and effective services that more closely meet their specific needs [41]. This consultation is critical to the sustainability and development of service provision for refugees, including regular screening, treatment and recording refugee’s progress in terms of health and justice services. It also enables service providers to better understand and work with community organisations who are providing informal services for refugees [13,22,23].

The research highlights some valuable work being undertaken by CSOs and some faith-based organisations in restoring hope in refugees. However, it is important to note that whilst these organisations are attempting to alleviate the negative life conditions of people in the communities they are serving, concerns have been raised regarding some faith-based organisations engaged in development work, and an emphasis sometimes on attempting to convert people to their own faith [42,43]. We therefore argue for a more nuanced and in-depth understanding of the role civil society and faith-based organisations play in the provision of health and justice provision that empowers refugee survivors of SGBV and torture in the context of international development [43].

- Need for Integrated Approach:

The research provides further evidence of the need to join up health and justice service responses for refugee survivors of SGBV and torture, which responds to changing refugee needs as determined through regular screening, treatment and reporting. The importance of integrated culturally and gender-sensitive approaches in both health and justice provision is borne out by our findings and builds on earlier work with conflict survivors which argued [20]:

“Sexual violence was experienced simultaneously as a violation of the survivors’ body and rights. It left the survivor in need of both a health and a justice response. As the two are connected in the experience of the survivor so they go hand in hand in terms of service responses required. We therefore argue that there is real value in promoting increased collaboration between local health and justice services”.

The current research extends this earlier work, to argue that improvements in refugee survivors’ dignity and resilience is dependent upon active engagement of refugees themselves, as well as combining formal and informal health and justice service responses, tackling structural reasons for SGBV and torture, as well as the provision of a culturally and gender-sensitive approach. Our research recommends an approach that integrates formal and informal health and justice services to meet refugees stated needs that includes listening to and responding to the needs and experiences of refugees in a way that builds their resilience and agency. 

### 4.1. Improving Health and Justice Responses

Our research recommendations are aimed at refugee organisations, government, non-government organisations, policy makers and international partners, with the expectation that they will further develop and implement these improvements where feasible. 

#### 4.1.1. Comprehensive Screening and Treatment for SGBV and Torture

- Organisations supporting refugees must ensure that there is routine and regular screening and documentation of SGBV and torture, that is sensitive to age, gender and access to justice.
- Service responses need to respond to refugee experiences in the country of origin, on route and within the country of settlement. This must include emotional and physical health treatment as well as access to formal and informal justice mechanisms [11,13].
- Development of effective support systems for service providers, including stronger links between health and justice providers and referral mechanisms. We support other research with South Sudanese refugees that supports developing accountability mechanisms that cater to all everyday experiences of injustice, as an important way of ensuring that refugee survivors of sexual violence and torture can seek redress, through interrupting the cyclical relationship between impunity and continued violence [11,13].
- Provision of the integrated services should include physical and psychological health and justice services, as well as education and viable livelihood opportunities. This approach would ensure that refugee’s feel validated, helping them to use their own resilience and agency to continue the process of recovery [14,15,23].

#### 4.1.2. Improved Health Responses

- Better clinic resources and effective referral processes including justice mechanisms for the recording and reporting of abuse.
- Responses need to ensure that there is adequate physical and psychological healthcare staff, and that services are extended, and sensitive to men in particular, who find it difficult to come forward to discuss their SGBV and torture experiences, due to stigma and shame and a lack of specialist medical and psycho-social service provision to respond to their specialist needs.
- The team also advises that CSOs be involved in informing services, to include traditional approaches to recovery building on refugees own skills [29], provision of psycho-social groups where required for refugees and their children, whilst also involving the healthcare teams and community-based organisations.

#### 4.1.3. Improved Justice Responses

Extending the use of mobile courts could assist in improving access to restorative justice and compensation at a local level; including the provision and resources of treatment for survivors of SGBV and torture, as well as an anti-discrimination provision with penalties for those who abuse refugees. Indeed, a recent report found the use of mobile courts in the Bidi Bidi refugee settlement proved successful for ensuring justice and enabled refugees to attend the sessions and learn more about Uganda law, which served as a deterrence in committing crimes [24]. We recommend the following:

- Increased knowledge regarding the South Sudanese customary court processes and challenging their structures to provide better gendered accountability for SGBV crimes with referral on to higher courts where required could be a positive way forward alongside survivor-focused local justice provision, contextualisation of violence and campaigning through community-based and women’s organisations [11,13].
- Strengthening community responses to SGBV and torture survivors whilst addressing the practices that re-victimise survivors, for example, forced marriage to perpetrators and abduction of young children for marriage, would help facilitate a long-term process of justice reform [11,13].
- The authors argue that it is imperative that governments and international partners, including service responders, listen to the voices of refugee survivors of SGBV and torture as a form of social justice through narration, and their view that integration between services is needed for recovery, restoration of dignity and building resilience.

#### 4.1.4. Further Research

We recommend that future gender and culturally sensitive research focusses on a systematic evaluation of the proposed integrated model of services for refugees and whether this impacts refugee recovery, resilience and dignity as well as tackling unaddressed trauma and the associated ‘cycles of violence’ evident [11,13,20]. It would be helpful to carry this out in different contexts and faith groups, including in urban locations, different community settings and various refugee settlements within Uganda and beyond.

## 5. Conclusions

The enormous influx of South Sudanese refugees into Uganda has reignited debates about the country’s refugee policy and, with it, discussions on the extent to which the “Ugandan Model” can be implemented in other countries in Africa and around the world. Given the growing numbers of refugees globally, and the momentum surrounding the global compact on refugees and the Comprehensive Refugee Response Framework (CRRF), these are vital discussions. In comparison to many other countries across the globe, not least economically richer parts of the world, Uganda’s willingness to host hundreds of thousands of refugees stands out as a positive example. Furthermore, the government has taken significant steps to allow for greater freedom of movement and access to work for refugees, again going against global trends. The positive aspects of Uganda’s approach, therefore, should be applauded [35].

Against this backdrop, it is vital that there is a clear understanding of the gap between rhetoric and reality, and some blind spots of the policy, especially in those areas in which the ostensibly progressive approach that the international community promotes may curtail access to rights. Firstly, international actors need to deliver on promises of significant financial support for meaningful positive changes to be achieved [5,10]. Funding was reported to be insufficient for the provision of adequate health, justice, education and vocational services, for instance, during an interview with a local government official it was stated:

“The Government of Uganda get no funding to support refugees and bad publicity ‘kills’ refugees. There is a lack of water points and infrastructure gaps including poor roads, offices, bridges and accommodation for staff, some of whom travel over 10 kilometres to get to work. Livelihoods are poor and the soil is infertile. Registered refugees survive on about £20 a month which is not sufficient.”

Secondly, the reality that the current situation in Uganda is a protracted refugee crisis needs to translate into a braver and more robust discussion for durable solutions [44]. Thirdly, whilst money is important, it cannot replace rigorous policy making and implementation that is more attuned to the lived daily realities and needs of refugees our research has highlighted [32]. Fourthly, the current research demonstrates the need for service integration through breaking down the traditional barriers between health, justice and livelihood services, as well as the need to regularly record and document SGBV and torture among different ages, genders, and abuse and torture experiences, alongside recourse to restorative justice. These should respond sensitively to the different lived experiences of refugee survivors and involve the relevant political and civil society based structures, both formal and informal, that represent them. 

Finally, we urge that organisations’ supporting refugees do their best to ensure there is provision of integrated, culturally and gender-sensitive services, including physical and psychological health and justice services, as well as livelihood opportunities such as the setting up of social enterprise groups, in order to increase the adaptation, wellbeing, resilience, dignity and recovery of refugee survivors of SGBV and torture [14,15,20,21,22,23,41,45,46]. 

## Figures and Tables

**Table 1:** Themes from the analysis of key stakeholder and refugee survivor interviews.

Main Themes	Sub-Themes
Nature of refugee sexual and gender-based violence (SGBV) and torture experiences	Sexual and Gender-Based Violence and torture
	Child abduction and marriage
	Family conflict and domestic violence
Service provision	Screening
	Health services
	Justice services
	Other services provided
Gender issues	Lack of gendered understanding
	Need for gender-informed specialist services
Involvement of civil society organisations (CSOs) and local non-governmental organisations (NGOs)	Provision of emotional support
	Instil hope
Improving health and justice responses	Comprehensive screening and treatment for SGBV and torture
	Improved health responses
	Improved justice responses
	Further research
